# Investigation of sports participation motivation in people with disabilities: a cross-sectional study of individuals with physical and hearing impairments

**DOI:** 10.1186/s13102-024-00846-0

**Published:** 2024-02-23

**Authors:** Erdem Ayyildiz, Dicle Aras, Fatma Hilal Yagin, Mehmet Gülü, Hakan Yapici, Reem Alwhaibi

**Affiliations:** 1 Department of Sports Management, Faculty of Sport Sciences, Tekirdag Namik Kemal University, Tekirdag, Turkey; 2 Ankara University Performance Analysis in Sports Application and Research Center, Department of Coaching Education, Faculty of Sport Sciences, Ankara University, Ankara, Turkey; 3 Department of Biostatistics and Medical Informatics, Faculty of Medicine, Inonu University, Malatya, 44280 Turkey; 4 Department of Sports Management, Faculty of Sport Sciences, Kirikkale University, Kirikkale, 71450 Turkey; 5 Department of Recreation, Faculty of Sport Sciences, Kirikkale University, Kirikkale, 71450 Turkey; 6 Department of Rehabilitation Sciences, College of Health and Rehabilitation Sciences, Princess Nourah bint Abdulrahman University, P.O. Box 84428, Riyadh, 11671 Saudi Arabia

**Keywords:** Auditory, Disabilities, Sports management, Sports psychology, Hearing

## Abstract

While reading the literature, it is seen that there are not enough studies on the motivation of disabled individuals to participate in sports. This study aims to examine the sports participation motivations of hearing impaired and physically disabled athletes. This study was a cross-sectional study. The research group of the study consists of physically and hearing-impaired individuals between the ages of 18–47. The participants of this research group consisted of 253 volunteer disabled individuals, 150 of whom were men and 103 of whom were women. Sports participation motivation scale was used for disabled individuals. The scale consists of 3 dimensions and is a 5-point Likert type. The results of the study showed that hearing-impaired people have a higher high school rate and physically person with disability have a higher bachelor’s degree rate, but the primary education rate did not change between hearing and physically person with disability. Physical activity participation differed between hearing and physically person with disability, and it was observed that hearing-impaired people participated in more physical activities. The level of well-being of the physically disabled was significantly better than the hearing impaired. As conclusion, it is observed that the people with the lowest motivation to participate in sports are primary school graduates and those with high welfare have a high motivation to participate in sports.

## Introduction

Motivation is an important driving force to take people to action on an issue [1, 2]. In order to motivate individuals for the action to be taken, it will be beneficial to analyze the basic motivation components well. Motivation has three basic components: goals, needs and effort. One of the main purposes of motivation is to motivate the person to achieve the goals. It is considered important to direct the person well so that motivation can be used for the benefit of the individual and contribute to success [3, 4].

What is important in evaluating motivation to participate in sports is to understand well what are the elements required to maintain physical activity. Because the individual’s expectation is very important in motivation to participate in sports. In order to learn this expectation better, it is necessary to identify motivation (such as intrinsic enjoyment or skill development and mastery) and external factors (such as rewards, better health, looking good) [5]. In another study, six factors (sports activity with friends, popularity, fitness and health, social status, sports activities, relaxation through sports) were identified for motivation to participate in sports [6]. In the study conducted in Turkey, it is emphasized that one of the important elements for disadvantaged individuals (elderly, disabled individuals, etc.) to exercise is to include them in social life [7]. In addition, in another study conducted in Turkey, “sports making people feel free” plays an important role in the motivation of disabled individuals to partisipten in sports, among the internal motivation factors of athletes [8].

More than 1 billion people with disabilities live worldwide. About 80% of them live in low- and middle-income countries. The sedentary lifestyle of these disabled individuals may cause higher health problems than non-disabled individuals [9, 10]. Studies show that school-aged children with disabilities tend to be less physically active than children without disabilities [11, 12]. Disabled individuals who are physically inactive during childhood and adolescence continue a sedentary lifestyle in their youth [13] and adulthood [14]. According to a study conducted in the United States, 56% of disabled individuals do not do sports [15].

Individuals with disabilities struggle with the fear of exclusion and the thought of failure caused by not being able to participate in social life by staying at home [16]. As a result of this situation, both physical and mental problems arise [17, 18]. As is known, sports have positive effects on physical, mental, and social health [19, 20]. Participating in different sports activities and trying sports branches in line with their abilities or interests helps disabled individuals protect both their physical and mental health [21, 22]. At the same time, it can support them in expanding their social environment and experiencing positive changes in their motivation levels [23]. For this reason, it is envisaged that disabled individuals can protect their physical, mental and social health by making sports and exercise a part of their lives [24].

Individuals may be physically capable of performing a specific task; however, if they have doubts about their own abilities, motivation has been evaluated as the most important problem that has a great impact on their performance [24]. This often emerges as a challenge that prevents people from starting their chosen sports activity [25]. In this context, individuals struggling with physical disabilities may need to make more effort and find sources of motivation to participate in sports [26]. Therefore, it is very important to examine the motivation levels of disabled athletes [25, 26]. Scientific studies show that the positive relationship between sports and health has been repeatedly confirmed [27–30]. For this reason, it is considered important to identify the factors that will motivate people with disabilities to participate in sports [31, 32]. Academic studies show that many reasons motivate people with disabilities to participate in sports. The most important of these are health and social interaction [33–36]. Motivating the individual appropriately will benefit him/her to do sports continuously [37]. In addition, some situations discourage participation in sports. Time constraints, cost, and lack of opportunity can be cited as important obstacles [35].

In recent years, there has been a significant awareness of governments in the participation of people with disabilities in physical activity [9, 10]. However, scientific studies show that the policies are insufficient and need to be improved. An example of this is the improvement of trainers for people with disabilities [37] to support the participation of people with disabilities in sports activities, facilities suitable for people with disabilities, transportation, and support for the costs of participating in physical activity [38–40] and the expansion of social policies [41]. should be improved. In addition, United Nations countries and associations; It has formal action plans to develop and promote the adoption of sports activities aimed at rehabilitating, educating, and improving the quality of life of people with disabilities [42]. It is considered important to act on the recommendations of scientific studies when implementing policies for the disabled [9–44].

Environmental factors are an important factor in motivating people with disabilities towards sports [45]. After the improvement of environmental factors, there is a need for qualified trainers to train people with disabilities [46]. After these opportunities are provided, disabled individuals will be more motivated to participate in sports. Research has been conducted to ensure that Dutch Paralympic athletes’ participation in sports is sustainable. These studies show that entertainment and relaxation, health, and physical fitness are important factors in making the participation of individuals with disabilities in competition and sports sustainable [47]. To ensure sustainability in participation in sports, there may be differences from country to country. There are many factors such as language, geographical conditions, and culture based on this difference [48].

According to the state data of August 2022, the number of people with at least one disability in Turkey is 2,511,950. 56% of this is male and 44% is female. While 179,867 of these disabled individuals are hearing impaired, 311,131 people are physically disabled. This information in the national data system shows that our sample group consists of approximately 490,998 people. When the literature was reviewed, no study was found on the motivation of hearing and physically disabled athletes to participate in sports in Turkey. Individuals with hearing and physical disabilities constitute approximately 6.2% of Turkey. For this reason, it is thought that it is of great importance to investigate the reasons for the motivation of people in this disability group to participate in sports. This study aims to examine the motivation of athletes with hearing and physical disabilities to participate in sports.

Hypotheses of the study:

1) H_1_: There is a significant difference in the motivation to participate in sports in person with disability depending on their disability situation.

2) H_1_: There is a significant difference in the motivation to participate in sports among person with disability according to gender.

3) H_1_: There is a significant difference in the motivation to participate in sports among person with disability according to welfare level.

## Materials and methods

### Study design and participants

This study was a cross-sectional study. The research group of the study consists of individuals with physical and hearing disabilities over the age of 18. The participants in this research group consisted of 258 voluntary disabled individuals, 150 of whom were male and 103 females. A total of 5 participants were excluded as follow (5 missing data). The inclusion criteria of the participants consist of members of associations and sports clubs for the disabled. With G*Power software (University of Duesseldorf, Duesseldorf, Germany, version 3.0.1), the independent sample t-test was used to calculate sample size and actual power (α = 0.05, power = 0.81, effect size = 0.36). The results revealed that with a sample size of 253 participants, the actual power was 81% [48].

### Procedures

Participants were informed about the measurement protocols and the purpose of the study. A form file was created for each of the participants who wanted to participate in the research voluntarily. Data were collected by giving a hand survey to hearing impaired and physically disabled athletes in disabled sports organizations in Turkey. The people participating in the research are physically disabled and hearing impaired individuals who actively engage in sports. The study was approved by the Kirikkale University Social and Human Sciences Ethics Committee (Date: 2022-06-18, No: 2022/06) and was conducted according to the principles stated in the Declaration of Helsinki. 5 participants who participated in the measurements were excluded from the study because they provided incomplete information.

### Measurements

#### Questionnaires

The first section contains demographic information. demographic information; gender, educational status, participation in physical activity, and welfare status.

In the second part of the measurement tool, the scale of motivation to participate in sports in individuals with disabilities was used. This scale was developed by Tekkursun et al. [8] for physically handicapped, hearing-impaired, and visually impaired individuals. The first factor of the scale of motivation to participate in sports in individuals with disabilities, the “Intrinsic Motivation Sub-Dimension” consists of 12 questions. The second factor “External Motivation Sub-Dimension” consists of 5 questions. The third factor, the “Lack of Motivation Sub-Dimension”, consists of 5 questions. The amotivation factor consists of inverse items The answer options for the items on the scale created with a 5-point Likert scale are arranged as “1 = Strongly Disagree”, “2 = Partially Disagree”, “3 = Moderately Agree”, “4 = Agree”, “5 = Strongly Agree”. Since the scores on the scale are between 1 and 5, the closer the propositions in the items are to 5, the higher the students’ motivation levels for participation in sports are; The closer it gets to 1, the lower it is considered. The values of the items representing the amotivation subscale were reversed during the scoring stage (1 = 5, 2 = 4, 3 = 3, 4 = 2, 5 = 1) [49].

#### Data analysis

The conformity of the quantitative variables to the normal distribution was examined using visual (histogram and probability plots) and analytical (Kolmogorov-Smirnov Test) methods. Descriptive statistics are expressed as the median, interquartile range (IQR) for non-normally distributed quantitative variables. Mann–Whitney U and Kruskal–Wallis H tests were used for comparisons of two or more groups regarding the variables that did not meet the parametric test assumptions, respectively. After the Kruskal–Wallis H test results, the Conover test was used for pairwise comparisons for universally significant variables. The effect size was calculated using the Cohen’s D. The magnitude of effect size was considered following the thresholds: Cohen suggested that d = 0.2 be considered a ‘small’ effect size, 0.5 represents a “medium” effect size, and 0.8 a “large” effect size (Cohen, 1988). Frequency (n) and percentage (%) values were calculated for qualitative variables. Pearson Chi-square tests were used to examine the relationships between qualitative variables. Relationships between variables were conducted using the Spearman rank correlation coefficient. In all results, *p*-value of < 0.05 was considered statistically significant. Statistical analyses were performed using SPSS 28.0 (IBM Corp., Armonk, NY, United States) package program.

## Results

The participants of this research group consisted of 253 volunteer disabled individuals, 150 of whom were men and 103 of whom were women, over the age of 18. The results of the study revealed that the rate of high school graduates was higher among the hearing impaired and the rate of bachelor’s degree graduates was higher among the physically disabled, but the rate of primary school graduates did not differ between the hearing and physically disabled. Participation in physical activity differed between the hearing and the physically disabled, with the hearing disabled participating in more physical activity. The welfare level of the physically disabled was significantly better than the hearing impaired (Table 1).

**Table 1:** Descriptive statistics on participants’ gender, education level, physical activity participation, and welfare level by disability status

Variable	Category	Disability situation		*p*-value
		Hearing Disabled	Physically Disabled	
		n(%)	n(%)	
**Gender**	**Male**	75 (50)	75 (50)	0.404
	**Female**	46 (44.7)	57 (55.3)	
**Educational Status**	**Primary Education**	7^a^ (58.3)	5^a^ (41.7)	**< 0.001**
	**High School**	97^a^ (63.0)	57^b^ (37.0)	
	**Bachelors Degree**	17^a^ (19.5)	70^b^ (80.5)	
**Physical Activity Participation**	**None**	16^a^ (44.4)	20^a^ (55.6)	**< 0.001**
	**Rarely**	44^a^ (66.7)	22^b^ (33.3)	
	**Sometimes**	45^a^ (36.0)	80^b^ (64.0)	
	**Always**	16^a^ (61.5)	10^a^ (38.5)	
**Welfare Level**	**Well**	3^a^ (12)	22^b^ (88)	**< 0.001**
	**Middle**	87^a^ (56.1)	68^b^ (43.9)	
	**Low**	31^a^ (42.5)	42^a^ (57.5)	

a, b: Different letter indicates a significant difference between group means

The changes in the mean scale scores of the participants according to their disability status are given in Table 2. There was a statistically significant difference between hearing and physically person with disability in terms of internal motivation (*p* = 0.014; ES = 0.312; large effect) and external motivation (*p* < 0.001; ES = 0.441; large effect) scores. Physically person with disability had significantly higher internal motivation and external motivation scores compared to hearing- person with disability. However, disability status was not significant for motivation score (*p* > 0.05).

**Table 2:** Changes in motivation scale scores according to disability status

Variable	Disability situation		*p*-value	ES
	Hearing Disabled	Physically Disabled		
	Median (IQR)	Median (IQR)		
**Inner Motivation**	47(10)	50(7)	**0.014**	0.312
**External Motivation**	18(1)	19(3)	**< 0.001**	0.441
**Lack of Motivation**	9(4)	9(2)	0.809	-

IQR: Interquartile Range; ES: effect size

The changes in the mean scale scores of the participants according to their educational status are given in Table 3. While there was no significant difference in terms of internal and external motivation, motivation scores were significantly higher in primary education (*p* < 0.001; ES = 0.732; large effect).

**Table 3:** Changes in motivation scale scores according to educational status

Variable	Educational Status			*p*-value	ES
	Primary education	High school	Bachelors degree		
	Median (IQR)	Median (IQR)	Median (IQR)		
**Inner Motivation**	37.5(27.5)	50(10)	46(21)	0.124	-
**External Motivation**	14(11)	19(1)	18(11)	0.194	-
**Lack of Motivation**	13.5^a^(10)	8^b^(4)	10^a^(8)	**< 0.001**	0.732

IQR: Interquartile Range; ES: effect size; Different letters indicate statistically significant differences (*p* ≤ 0.05). a, b: Different letter indicates a significant difference between group means

Gender was a significant factor in motivation scale scores. Intrinsic (*p* < 0.001; ES = 0.411; large effect) and extrinsic (*p* < 0.001; ES = 0.510; large effect) motivation scores were significantly higher in men, whereas the motivation score was higher in women compared to men (*p* = 0.003; ES = 0.369; large effect) **(**Table 4**).**

**Table 4:** Changes in motivation scale scores according to gender

Variable	Gender		*p*-value	ES
	Male	Female		
	Median (IQR)	Median (IQR)		
**Inner Motivation**	50.5(7)	46(21)	**< 0.001**	0.411
**External Motivation**	19(1)	18(7)	**< 0.001**	0.510
**Lack of Motivation**	9(2)	9(9)	**< 0.003**	0.369

IQR: Interquartile Range; ES: effect size

The changes in the motivation scale scores of the participants according to their welfare level are given in Table 5. When Table 5 is analyzed, welfare level is an important factor for motivation scale scores. Those with low welfare levels had significantly higher internal motivation scores (*p* < 0.001; ES = 0.893; large effect). In addition, the external motivation score was significantly higher in those with low welfare compared to those with high welfare (*p* < 0.05), whereas there was no difference in the external motivation score between those with moderate and high welfare (*p* > 0.05). Moreover, it was determined that those with well welfare results had higher motivation scores.

**Table 5:** Changes in motivation scale scores according to welfare level

Variable	Welfare Level			*p*-value	ES
	Well	Middle	Low		
	Median (IQR)	Median (IQR)	Median (IQR)		
**Inner Motivation**	29^a^ (3)	47^b^ (10)	51^c^ (5)	**< 0.001**	0.893
**External Motivation**	12^a^ (0)	19^b^ (1)	19^b^ (3)	**< 0.001**	0.699
**Lack of motivation**	17^a^ (0)	9^b^ (4)	8^c^ (1)	**< 0.001**	0.981

IQR: Interquartile Range; ES: effect size; Different letters indicate statistically significant differences (*p* ≤ 0.05). a, b, c: Different letter indicates a significant difference between group means

No significant correlation was found between the motivation scale scores and age in the hearing impaired (*p* > 0.05). In addition, it was determined that there was a high level of positive correlation between the inner motivation score and the external motivation score, while there was a strong negative relationship between the inner motivation score and the external motivation score, and the amotivation score. It was observed that the external motivation score decreased with increasing age in the physically disabled. Moreover, a positive relationship was determined between the inner motivation score and the external motivation score in physically person with disability, while a strong negative relationship was determined between the inner motivation score and the amotivation score. In physically disabled individuals, as the external motivation score increased, the amotivation score decreased (Table 6).

**Table 6:** The relationship between the ages of the participants and their motivation scale scores

Disability situation	Variable	Statistics	Age(years)	Inner Motivation	External Motivation	Lack of Motivation
**Hearing Disabled**	**Age (years)**	rho	1.000	-0.046	-0.060	-0.040
		*p*-value		0.618	0.513	0.664
	**Inner Motivation**	rho		1.000	0.869	-0.904
		*p*-value			**< 0.001**	**< 0.001**
	**External Motivation**	rho			1.000	-0.904
		*p*-value				**< 0.001**
	**Lack of Motivation**	rho				1.000
		*p*-value				
**Physically Disabled**	**Age (years)**	rho	1.000	-0.042	-0.261	0.125
		*p*-value		0.635	**0.003**	0.153
	**Inner Motivation**	rho		1.000	0.313	-0.762
		*p*-value			**< 0.001**	**< 0.001**
	**External Motivation**	rho			1.000	-0.434
		*p*-value				**< 0.001**
	**Lack of Motivation**	rho				1.000
		*p*-value				

## Discussion

It has been reported that participation in sports has positive contributions not only to the physical dimension but also to the psychological and social dimensions of health not only in adults but also in children and youth [50]. However, very few studies have been found in the literature examining the motivation of physically disabled and deaf athletes [51, 52]. The first step in overcoming these barriers is to identify them. Therefore, this study purposed to investigate the motivations of physically disabled and deaf athletes in Turkey to participate in sports. For this purpose, some demographic information of the athletes was collected and the athletes were asked to fill the Sports Participation Motivation Questionnaire. The inner motivation, external motivation, and demotivation scores, and their relationship with age, gender, educational status, physical activity participation, and welfare level were determined. Significant differences were observed in many of the descriptive parameters. While determining these parameters, the literature was scanned. In the literature review, factors affecting sports participation include gender, educational status [53], participation in physical activity [54] age [55–57], and welfare level [27] seem to be important. It was observed that most of the bachelor’s graduates were composed of physical disabled groups and high school graduates consisted of deaf athletes. Participation in physical activity was more in deaf athletes. Considering the positive effects of participating in physical activity on all parameters of health [58]. İt is understood that studies should be carried out to increase the participation of people with physical disabilities.

When the motivation to participate in sports among hearing and hearing-impaired individuals was examined, no significant difference was found in the education variable [59]. Another study supports us [60]. The main reason for this situation is thought to be the level of access to the elements that will motivate disabled individuals, rather than education in motivation to participate in sports.

In a study, it was determined that the motivation of physically person with disability to participate in sports was significantly higher than hearing-impaired individuals [8]. This situation is parallel to our study. When the results are examined according to disability status, a high level of inner motivation score (*p* = 0.014), and external motivation score (*p* < 0.001) were observed in physically disabled athletes. These results indicate that being deaf affects the motivation more negatively. However, it was found that demotivation, which is referred to as a lack of motivation, did not differ according to the disability condition. Considering that 15.6% of the world’s population is included in any disability category [61] approximately 40% of people with disabilities have a chronic illness in-dependent of their disability [62]. The results of the research are important for the development of new strategies on the subject. Studies suggest that although disability leads people to a sedentary life, a physically active life that these people will achieve through sports will have positive physical and social effects [63]. However, there are still social barriers that prevent the participation and continuity of people with disabilities in sports [64], and 40% in the UK and 44.3% in the USA of people with disabilities lead a physically inactive life [65]. While there are studies finding that the motivation to participate in sports is significantly higher in individuals with physical disabilities [59, 66], there are studies in which there is no significant difference [67]. The main reason for this situation is thought to be whether there are opportunities in the environment where individuals are located that will affect their motivation to participate in sports, depending on their disability, rather than their disability. It is thought that the reason why there is no significant difference in the motivation of the participants in our study to participate in sports is that appropriate conditions are provided according to the disability group.

It has been determined that there is no significant difference in the motivation of hearing impaired athletes to participate in sports on the gender variable [68]. Internal and external motivation scores of men were found higher than women (*p* < 0.001), and women’s demotivation scores were higher than men (*p* = 0.003).

It was concluded that there is no significant difference in the motivation to participate in sports in terms of the gender variable due to the equal sports opportunities for men and women [69–71]. In addition, similar results were obtained in a study conducted in Turkey [72], where women’s motivation to participate in sports was higher than men in China [73]. The results show that there are more opportunities in Turkey that can positively affect men’s motivation to participate in sports. It is thought that one of the reasons for the low motivation of women to participate in sports in Turkey is cultural factors.

This study has revealed important information about motivation and welfare level in disabled athletes. The inner motivation was found to be higher in low welfare group significantly than medium and well level of welfare groups (*p* < 0.001 with a large effect size of 0.893). Similar results were observed also in external motivation. The low welfare group showed the highest score than high welfare group (*p* < 0.05). However, there was no difference between medium and high-level welfare groups. Similarly, demotivation score of the well-welfare group was observed to be highest.

The level of well-being is important in the motivation for sports participation in disabled individuals. For this reason, people with low welfare levels should be supported by state policies [74]. It is thought that supporting disabled individuals with low welfare levels will significantly increase their motivation to participate in sports [70, 71]. In Turkey, local governments and the Ministry of Sports provide important opportunities to disabled individuals. In particular, the economic support given according to the level of welfare may be the reason why people with a low level of welfare have a significantly higher effect on the motivation to participate in sports.

Studies show that disability and age are important in motivation to participate in sports [55–57]. One of the biggest problems of disabled individuals in participating in sports is that their health problems increase with increasing age [75]. It is observed that hearing-impaired people have fewer physical health problems and physically person with disability suffer from more diseases [76]. This information supports our work. It is thought that hearing-impaired people suffer from fewer diseases and therefore there is no relationship between age and motivation to participate in sports. However, this is the opposite for physically person with disability. In other words, diseases increase as age increases in physically person with disability, and it is thought that the motivation to participate in sports decreases for this reason.

## Conclusions

According to the results of the current research, these problems can be listed as follows: (1) The motivation level of both disability groups, especially the deaf athletes, should be increased, (2) In-creasing the education level of person with disability causes them to understand the relation-ship between physical activity and health, and thus increases their participation in sports, (3) Gender has a significant impact on motivation, and efforts should be made to increase the participation and persistence of especially women with disabilities in sports, (4) It is considered important that the positive effects of sports on health are presented independently of the socio-economic structure in order to prevent the decrease in motivation in participation in sports as the income level increases, (5) In order to prevent the decrease in the external motivation of people with physical disabilities as their age increases, it is necessary to better explain the positive effects of high-level physical activity levels, especially in later ages.

The practical implications of the study show that local governments need to work to increase the motivation of disabled individuals to participate in sports. It is considered important that both academics and politicians work in a coordinated manner to increase academic studies on disabled individuals and disseminate sports culture.

### Limitations

A limitation of this study is its cross-sectional methodology. Unfortunately, we were unable to collect longitudinal data in this first part of the study. Future research should include a longitudinal method to better represent the developmental course of this proposed disorder. Another limitation is that the participants of this study are limited to Turkish hearing-impaired and physically disabled individuals over the age of 18. The generalizability of the findings may be limited due to the small sample size.

## Data Availability

Data are available for research purposes upon reasonable request to the corresponding author.

## References

[1] Ntoumanis N, Ng JY, Prestwich A, Quested E, Hancox JE, Thøgersen-Ntoumani C, Williams GC (2021). A meta-analysis of self-determination theory-informed intervention studies in the health domain: effects on motivation, health behavior, physical, and psychological health. Health Psychol Rev.

[2] Schunk DH, DiBenedetto MK (2020). Motivation and social cognitive theory. Contemp Educational psycholo-gy.

[3] Kharkov VB, Kovač VB. (2016). The self-expression motivational system. *Basic Motivation and 4Human Behaviour: Control, Affiliation and Self-expression*, 133–168.

[4] Kovac VB. Basic motivation and human behaviour: control, affiliation and self-expression. Springer; 2016.

[5] Moradi J, DANA BAHRAMIA, Amir (2020). Motivation for participation in sports based on athletes in team and individual sports. Phys Cult Sport Stud Res.

[6] Kondric M (2013). Participation motivation and student’s physical activity among sport students in three countries. J Sports Sci Med.

[7] Gülü M, Ayyildiz E (2022). Effect of the COVID-19 pandemic on barriers to middle-aged adults’ participation in physical activity in Turkey: a cross-sectional study. J Men’s Health.

[8] Demir GönülTekkurşun, İlhan (2020). Levent. Engelli sporcularda spora katılım motivasyonu. Ankara Üniversitesi Eğitim Bilimleri Fakültesi Özel Eğitim Dergisi.

[9] Rimmer JH, Marques AC (2012). Physical activity for people with disabilities. Lancet.

[10] Martin Ginis KA, Van Der Ploeg HP, Foster C, Lai B, Mcbride CB, Ng K, Pratt M, Shirazipour CH, Smith B, Vásquez PM, Heath GW (2021). Participation of people living with disabilities in physical activity: a global perspective. Lancet.

[11] Pan C-Y, Frey GC, Bar-Or O, Longmuir P (2005). Concordance of physical activity among parents and youth with physical disabilities. J Dev Phys Disabil.

[12] Kim Y, Conners RT, Hart PD, Kang YS, Kang M (2013). Association of physical activity and body mass index with metabolic syndrome among U.S. adolescents with disabilities. Disabil Health J.

[13] Bandini L, Danielson M, Esposito LE, Foley JT, Fox MH, Frey GC, Fleming RK, Krahn G, Must A, Porretta DL, Rodgers AB, Stanish H, Urv T, Vogel LC, Humphries K (2015). Obesity in children with developmental and/or physical disabilities. Disabil Health J.

[14] Rimmer JA, Rowland JL (2008). Physical activity for youth with disabilities: a critical need in an underserved popula-tion. Dev Neurorehabil.

[15] Health USDO, Coordinating HSOH (2016). U.S. Department of Health and Human Services Oral Health Strategic Framework, 2014–2017. Public Health Rep.

[16] Stough LM, Sharp AN, Resch JA, Decker C, Wilker N (2016). Barriers to the long-term recovery of individuals with disabilities following a disaster. Disasters.

[17] Forber-Pratt AJ, Lyew DA, Mueller C, Samples LB (2017). Disability identity development: a systematic review of the literature. Rehabil Psychol.

[18] Ugurlu D, Emlek B, Yapici H, Gok O, Unver R, Sofuoglu M, Ayan S, Yılmaz A. Examination of physical activity levels of Turkish adults living in Rural and Urban Areas. Volume 1. Journal of Exercise Science & Physıcal Activity Re-views; 2023. pp. 12–23. 1[CrossRef].

[19] Molton IR, Yorkston KM (2017). Growing older with a physical disability: a special application of the successful Ag-ing paradigm. J Gerontol B Psychol Sci Soc Sci.

[20] Yapici H, Yagin, Hilal F, Emlek B, Uca E, Ayyıldız E, Ahmedov F (2023). Rintaugu, Elijah Gtonga., and Badri AL-Mhanna, Samer. Examining barriers to participation in physical activity: a study of adults. J Exerc Sci Physıcal Activity Reviews.

[21] Abes ES, Wallace MM (2018). People see me, but they don’t see me: an intersectional study of College Students with Physical Disabilities. J Coll Student Dev.

[22] Bunbury S (2019). Unconscious bias and the medical model: how the social model may hold the key to transformative think-ing about disability discrimination. Int J Discrimination Law.

[23] Wright A, Roberts R, Bowman G, Crettenden A (2019). Barriers and facilitators to physical activity participation for children with physical disability: comparing and contrasting the views of children, young people, and their clinicians. Disabil Rehabil.

[24] Brittain I, Biscaia R, Gérard S (2020). Ableism as a regulator of social practice and disabled peoples’ self-determination to participate in sport and physical activity. Leisure Stud.

[25] Allan V, Smith B, Côté J, Ginis KAM, Latimer-Cheung AE (2018). Narratives of participation among individuals with physical disabilities: a life-course analysis of athletes’ experiences and development in parasport. Psychol Sport Exerc.

[26] Hall SS, Macmichael J, Turner A, Mills DS (2017). A survey of the impact of owning a service dog on quality of life for individuals with physical and hearing disability: a pilot study. Health Qual Life Outcomes.

[27] Gülü M, Ayyıldız E. Effect of the COVID-19 pandemic on barriers to middle-aged adults’ participation in physical activity in Turkey: a cross-sectional study. J Mens Health. 2022. [CrossRef].

[28] Reiner M, Niermann C, Jekauc D, Woll A (2013). Long-term health benefits of physical activity—a systematic review of lon-gitudinal studies. BMC Public Health.

[29] Schmidt SCE, Tittlbach S, Bös K, Woll A. Different types of Physical Activity and Fitness and Health in adults: an 18-Year longitudinal study. Biomed Res Int 1785217, 2017. [CrossRef]. 10.1155/2017/1785217 PMC5390631 28466006

[30] Klemm K, Krell-Roesch J, De Clerck IL, Brehm W, Boes K (2021). Health-related fitness in adults from eight Euro-Pean Countries—An analysis based on data from the European Fitness Badge. Front Physiol.

[31] Apelmo E. What is the problem? Dis/ability in Swedish physical education teacher education syllabi. Sport Educ Soc. 2021, 1–14. [CrossRef].

[32] Mundhenke L, Hermansson L, Sjöqvist NB (2010). Experiences of Swedish children with disabilities: activities and so-cial support in daily life. Scand J Occup Ther.

[33] Bigby C, Anderson S, Cameron N (2018). Identifying conceptualizations and theories of change embedded in interven-tions to facilitate community participation for people with intellectual disabilities: a scoping review. J Appl Res Intellect Disabil.

[34] Mcloughlin G, Fecske CW, Castaneda Y, Gwin C, Graber K (2017). Sport participation for elite athletes with physical disabilities: motivations, barriers, and facilitators. Adapted Phys Activity Q.

[35] Swanson SR, Colwell T, Zhao Y (2008). Motives for participation and importance of social support for athletes with physical disabilities. J Clin Sport Psychol.

[36] Thorn SH, Pittman A, Myers RE, Slaughter C (2009). Enhancing community integration and inclusion for people with intellectual disabilities. Res Dev Disorders.

[37] Litwic-Kaminska K, Izdebski P (2012). The concept, self-esteem of health, health behavior and physical activity level in early adulthood. Pol J Sports Med.

[38] McGarty AM, Melville CA (2018). Parental perceptions of facilitators and barriers to physical activity for children with intellectual disabilities: a mixed methods systematic review. Res Dev Disabil.

[39] Wilson NC, Khoo S (2013). Benefits and barriers to sports participation for athletes with disabilities: the case of malay-sia. Disabil Soc.

[40] Thomson A, Bridges S, Corrins B, Pham J, White C, Buchanan A (2018). The impact of physical activity and sport programs on community participation for people with intellectual disability: a systematic review. J Intellect amp; Dev Disabil.

[41] Tekkurşun GD, İlhan LE, Esentürk O, Kan A (2018). Sport Participation Motivation Scale in Disabled individuals (Eskmö): Validity and Reliability Study. Spormetre.

[42] Beacom A, French L, Kendall S (2016). Reframing impairment? Continuity and change in media representations of disa-bility through the Paralympic games. Int J Sport Communication.

[43] Oh A, So WY. Assessing the needs of people with disabilities for physical activities and sports in South Korea. Healthcare. MDPI; 2022. p. 265. [CrossRef]. 10.3390/healthcare10020265 PMC8872264 35206880

[44] Wedgwood N (2014). Hahn versus Guttmann: revisiting ‘sports and the political movement of disabled persons’. Disabil Soc.

[45] Saebu M, Sørensen M (2011). Factors associated with physical activity among young adults with a disabil-ity. Scand J Med Sci Sports.

[46] Vanlandewijck Y (2006). Sport science in the paralympic movement. J Rehabil Res Dev.

[47] Jaarsma EA, Geertzen JH, de Jong R, Dijkstra PU, Dekker R (2014). Barriers and facilitators of sports in Dutch para-lympic athletes: an explorative study. Scandinavian J Med amp; Sci Sports.

[48] Hampton R, Toombs M (2013). Indigenous australians and Health: the Wombat in the room.

[49] Faul F, Erdfelder E, Lang AG, Buchner A (2007). G* power 3: a flexible statistical power analysis program for the social, behavioral, and biomedical sciences. Behav Res Methods.

[50] Eime RM, Young JA, Harvey JT, Charity MJ, Payne WR (2013). A systematic review of the psychological and social benefits of participation in sport for children and adolescents: informing development of a conceptual model of health through sport. Int J Behav Nutr Phys Activity.

[51] Meric S, Turay O (2020). The relationship between the motivation of wheelchair basketball players to participate in sports and quality of life. Yüzüncü Yıl Univ J Inst Social Sci.

[52] Emamvirdi R, Ilhan ARH, Colakoglu L (2020). Psychological flexibility and motivation to participate in sports in physically disabled athletes. J Phys Educ Sport Sci.

[53] Yüksel M. Differences in popular sports branches on the axes of gender and education level. Electron Turkish Stud, 2014, 9(5).

[54] Topsaç M, Bişğin H. Investigation of the physical activity levels of disabled students studying at the university. Dumlupınar Univ J Social Sci, 2013, (40).

[55] Adams D. Sports-based intervention as a tool for social inclusion? In Proceedings of International Conference on Special Education. 2017, (Vol. 2). 691–699.

[56] McConkey R, Dowling S, Hassan D, Menke S (2013). Promoting social inclusion through unified sports for youth with intel-lectual disabilities: a five-nation study. J Intellect Disabil Res.

[57] Simplican SC, Leader G, Kosciulek J, Leahy M (2015). Defining social inclusion of people with intellectual and develop-mental disabilities: an ecological model of social networks and community participation. Res Dev Disabil.

[58] Physical Activity Guidelines Advisory Committee. Physical Activity Guidelines Advisory Committee Scientific Report., 2018. Washington, DC: U.S. Department of Health and Human Services; 2018.

[59] Yilmaz A, Kirimoglu H, Mirze F (2020). Examining the sports participation motivation levels of physically disabled and hearing impaired athletes. Int J Appl Exerc Physiol.

[60] Abakay U. The relationship between the footballer-coach communication and the achievement motivation of the foot-ballers in different statuses. Unpublished dissertation, Gazi University, 2010.

[61] World Health (2011). Organization World report on disability.

[62] Carroll DD, Courtney-Long EA, Stevens AC, Sloan ML, Lullo C, Visser SN et al. Vital signs: disability and physical activity - United States, 2009–2012. Morbidity and mortality weekly report, 2014, 63(18), 407. PMID: 24807240; PMCID: PMC5779402. PMC5779402 24807240

[63] Weiler R, Van Mechelen W, Fuller C, Verhagen E (2016). Sport injuries sustained by athletes with disability: a systematic review. Sports Med.

[64] Rimmer JH, Riley B, Wang E, Rauworth A, Jurkowski J (2004). Physical activity participation among persons with disabilities: barriers and facilitators. Am J Prev Med.

[65] Aitchison B, Rushton AB, Martin P, Barr M, Soundy A, Heneghan NR (2022). Experiences and perceived health ben-efits of people with disabilities participating in sport: a systematic review and narrative synthesis. J Disabil Health.

[66] Shihui C, Jin W, Mei J, On LK. Motivation of sport participation in elite athletes with physical disabilities in mainland China. Asian J Exerc Sports Sci, 2007, 4(1).

[67] Pittet I, Berchtold A, Akré C, Michaud PA, Suris JC (2009). Sports practice among adolescents with chronic health con-ditions. Arch Pediatr Adolesc Med.

[68] Demir GT, İlhan EK (2019). Sports participation motivation: a study on visually impaired athletes a research. Gaziantep Univ J Sport Sci.

[69] Baikoglu S, Yesilkaya B (2020). Analyzing sports participation motivations of students with hearing impair-ment–Istanbul City Example. Int Educ Stud.

[70] Koivula N (1999). Sport participation: differences in motivation and actual due to gender typing. J Sport Behav.

[71] Požėrienė J, Adomaitienė R, Ostasevičienė V, Rėklaitienė D, Kragnienė I. Sport participation motivation of athletes with intellectual disabilities. Baltic J Sport Health Sci, 2008, 3(70). [CrossRef].

[72] Kızar O, Demir GT, Genç H. Examination of the effect of national and amateur disabled athletes’disabilty types on sports participation motivation. Eur J Phys Educ Sport Sci, 2021, 6(12). [CrossRef].

[73] Sit CH, Lindner KJ, Sherrill C (2002). Sport participation of Hong Kong Chinese children with disabilities in special schools. Adapted Phys Activity Q.

[74] Perreault S (2007). A test of sellf-determination theory with wheelchair basketball players with and without disability. Adapted Phys Activity Q.

[75] Hassett L, Shields N, Cole J, Owen K, Sherrington C. Comparisons of leisure-time physical activity participation by adults with and without a disability: results of an Australian cross-sectional national survey. BMJ open Sport Exerc Med, 2021, 7(1), e000991. [CrossRef]. 10.1136/bmjsem-2020-000991 PMC7797250 33489311

[76] Hill-Briggs F, Dial JG, Morere DA, Joyce A (2007). Neuropsychological assessment of persons with physical disability, visual impairment or blindness, and hearing impairment or deafness. Arch Clin Neuropsychol.

